# Larva of greater wax moth *Galleria mellonella* is a suitable alternative host for the fish pathogen *Francisella noatunensis* subsp. *orientalis*

**DOI:** 10.1186/s12866-020-1695-0

**Published:** 2020-01-09

**Authors:** Winarti Achmad Sarmin Djainal, Khalid Shahin, Matthijs Metselaar, Alexandra Adams, Andrew P. Desbois

**Affiliations:** 1 Institute of Aquaculture, Faculty of Natural Sciences, University of Stirling, Stirling, FK9 4LA UK; 2 Polytechnic State of Pontianak, Jalan Jenderal Ahmad Yani, Pontianak, Kalimantan Barat 78124 Indonesia; 3 Present address: Department of Medicine and Epidemiology, School of Veterinary Medicine, University of California, Davis, CA 95616 USA; 4 Benchmark Animal Health Ltd, Edinburgh, EH26 0BB UK

**Keywords:** Alternative model, Aquaculture, Piscine francisellosis, *Oreochromis niloticus*, Red Nile tilapia

## Abstract

**Background:**

*Francisella noatunensis* subsp. *orientalis* (*Fno*) is the etiological agent of francisellosis in cultured warm water fish, such as tilapia. Antibiotics are administered to treat the disease but a better understanding of *Fno* infection biology will inform improved treatment and prevention measures. However, studies with native hosts are costly and considerable benefits would derive from access to a practical alternative host. Here, larvae of *Galleria mellonella* were assessed for suitability to study *Fno* virulence.

**Results:**

Larvae were killed by *Fno* in a dose-dependent manner but the insects could be rescued from lethal doses of bacteria by antibiotic therapy. Infection progression was assessed by histopathology (haematoxylin and eosin staining, Gram Twort and immunohistochemistry) and enumeration of bacteria recovered from the larval haemolymph on selective agar. *Fno* was phagocytosed and could survive intracellularly, which is consistent with observations in fish. Virulence of five *Fno* isolates showed strong agreement between *G. mellonella* and red Nile tilapia hosts.

**Conclusions:**

This study shows that an alternative host, *G. mellonella*, can be applied to understand *Fno* infections, which will assist efforts to identify solutions to piscine francisellosis thus securing the livelihoods of tilapia farmers worldwide and ensuring the production of this important food source.

## Background

Piscine francisellosis is a global disease caused by the bacterium *Francisella noatunensis*, with *F. noatunensis* subsp. *orientalis* (*Fno*) infecting warm water fish and *F. noatunensis* subsp. *noatunensis* (*Fnn*) affecting cold water species [1]. Once inside a host, like other *Francisella* spp. pathogens, *F. noatunensis* survives and replicates in host cells, particularly phagocytes such as monocytes, macrophages, neutrophils and phagocytic B-cells [2–11]. *Fno* is a particular concern for tilapia producers as it can cause up to 95% mortality [12, 13] and diagnosis of francisellosis is challenging, especially due to difficulties isolating this fastidious bacterium and the presence of other pathogens, which may have led to underreporting of the problem [1]. Current therapy relies on antibiotics and no safe and effective commercial vaccine is available, though there is progress towards its development [14].

Relatively little is known of the infection biology of *Fno* and a deeper fundamental understanding of virulence and pathogenicity may inform new and improved treatments, prevention measures and farm management practices. To this end, experimental studies have been performed in the native fish hosts and, though these trials have extended our knowledge of francisellosis, such an approach is costly, requires specialist infrastructure such as aquaria, raises ethical questions and can be constrained by legal statutes. Moreover, a lack of access to animals of the right age and size can also impact on these experiments. Hence, more practical alternative hosts that offer insights into the biology of *F. noatunensis* infections have been explored including zebrafish and their embryos [6, 15]; however, this fish model suffers similar drawbacks to native hosts and best practice in research seeks adherence to the principles of the 3Rs, i.e. the replacement, reduction and refinement of the use of animals in experiments [16, 17].

As a result, non-vertebrate alternative hosts have been pursued as a way to study bacterial pathogens of fish, and this has led to investigations in the slime mould amoeba *Dictyostelium discoideum* [18, 19], the freshwater ciliate *Tetrahymena thermophile* [20], the nematode *Caenorhabditis elegans* [21], the crustacean *Artemia franciscana* [22], and the insect *Galleria mellonella* [23]. Of these, the larva of *G. mellonella* has considerable practical and biological benefits [24, 25], which has seen it used widely to study human pathogens, including the relatively low costs associated with sourcing, storage and disposal; ease of acquiring the skills needed to perform experiments; ability to deliver precise doses of a pathogen, examine pathology and perform studies at different temperatures; and the strong correlation in the virulence of pathogens in *G. mellonella* and vertebrate hosts [26, 27]. Indeed, an earlier study demonstrated the virulence of 11 *Vibrio anguillarum* isolates correlated strongly between the native Atlantic salmon (*Salmo salar*) host and the *G. mellonella* alternative host [23]. The insect immune system shares structural and functional characteristics with vertebrates but lacks the adaptive response; however, this still permits valuable insight into pathogen interactions with innate defences [28, 29]. Fish rely on the innate arm of immunity to defend against pathogens and similar humoral and cellular processes are present in fish and insects with respect to pathogen recognition; inducible production of lysozyme, antimicrobial peptides, reactive intermediates of oxygen and nitrogen species; phagocytosis of invading microbes; and signalling cascades that regulate coagulation and melanisation [28–35]. More recently, the *G. mellonella* genome has been sequenced which allows for even better understanding of host-pathogen interactions at the molecular level and can further the interpretation of findings with greater biological relevance [36]. Importantly, pathogens respond similarly to conditions *in vivo* when evading host defences and exploiting host tissues through conserved mechanisms of virulence, including cell adhesion and invasion, antioxidant protection measures, metal ion uptake, secretion systems, and toxin and enzyme production [23, 26, 27, 37–39]. Of note, *G. mellonella* has been used as an alternative host to understand infections by other *Francisella* spp., including the human pathogens *Francisella hispaniensis* [40], *Francisella novicida* [40], *Francisella philomiragia* [41] and *Francisella tularensis* [42, 43].

Therefore, *G. mellonella* may prove suitable for studying the virulence and pathogenicity of *Fno*; however, first it is necessary to confirm that an infection occurs and virulence reflects that observed in the native host, including with respect to conserved mechanisms of virulence. Thus, the aim of the present study was to assess the suitability of *G. mellonella* as an alternative model for studying the virulence and pathogenicity of *Fno*.

## Results

### Effect of temperature on *G. mellonella* survival after injection with *Fno*

**Fig. 1:**
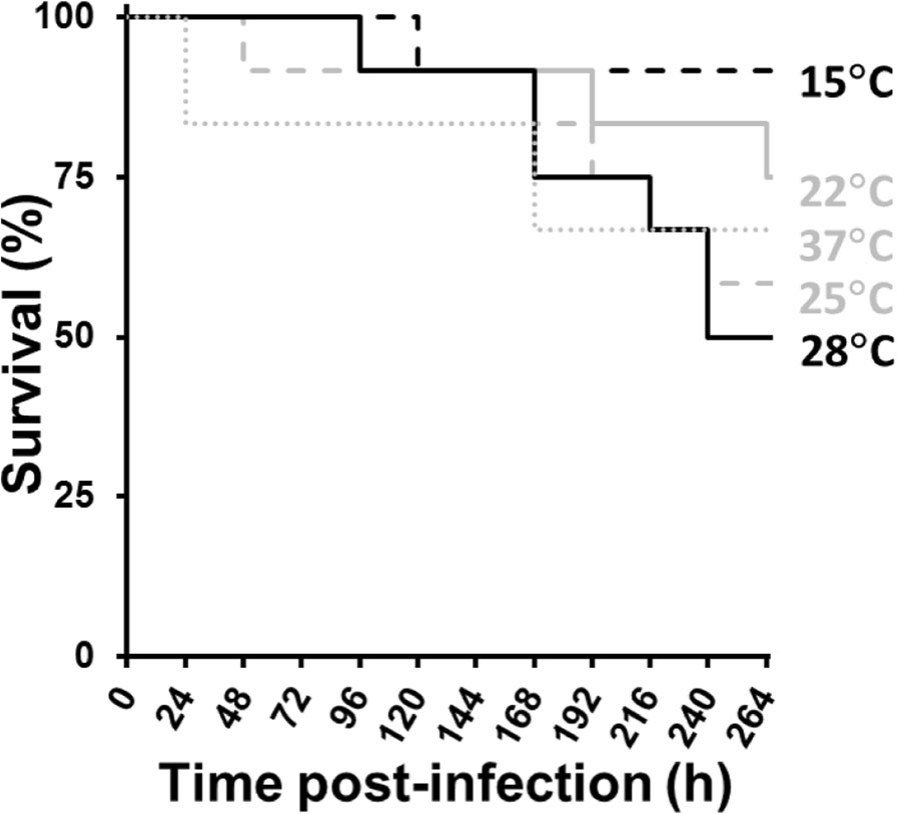
Effect of temperature on *Galleria mellonella* larva survival after injection with *Fno*. Kaplan-Meier plot of *G. mellonella* larva survival during 264 h after injection with *Fno* STIR-GUS-F2f7 at 1 × 10^9^ CFU/mL and incubated at 15, 22, 25, 28 and 37 °C, showing that the group of larvae incubated at 28 °C had lowest survival; survival was 100% in the unmanipulated and PBS only control groups at all temperatures (data not shown). *n* = 12

In the initial experiment to determine the effect of temperature on survival of *G. mellonella* larvae after injection with ca. 1 × 10^9^ colony-forming units (CFU)/mL of *Fno* STIR-GUS-F2f7 (isolated from Nile tilapia, *Oreochromis niloticus* [10]), the group of larvae incubated at 28 °C appeared to have lowest survival while the group kept at 15 °C had greatest survival (Fig. 1), so in all subsequent experiments larvae were incubated at 28 °C. Larvae injected with phosphate-buffered saline (PBS) only showed little change during the experiment from the usual cream body colour, but those injected with bacteria typically started to darken within hours due to melanisation, particularly along the dorsal midline, and the body became increasingly darkened up to death or the end of the experiment.

### Virulence of five isolates of *Fno* in *G. mellonella*

**Fig. 2:**
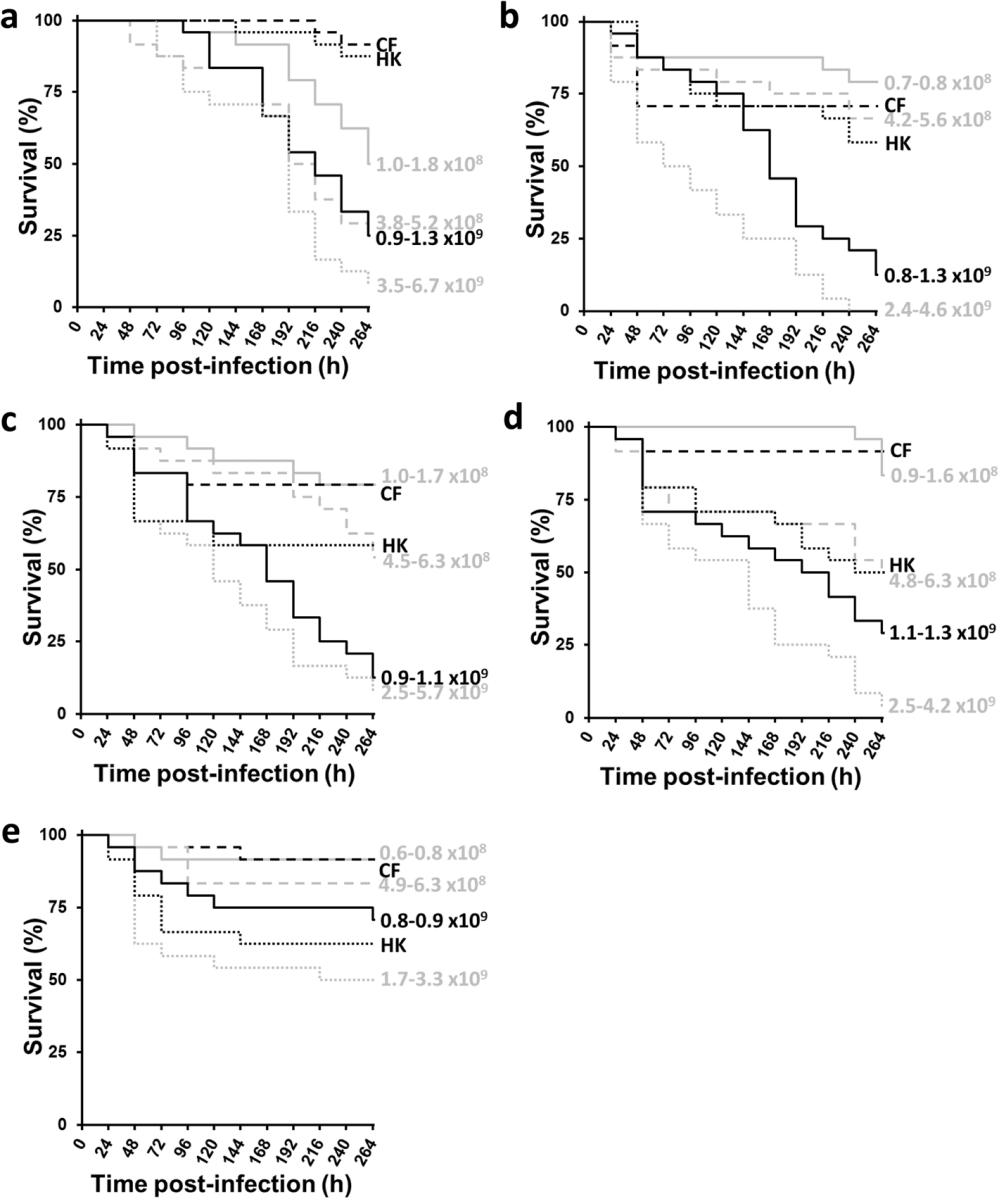
Effect of different doses of five *Fno* isolates on *Galleria mellonella* larva survival. Kaplan-Meier plots of *G. mellonella* larva survival during 264 h at 28 °C after injection of ca. 1 × 10^8^, 5 × 10^8^, 1 × 10^9^ and 5 × 10^9^ CFU/mL of (**a**) *Fno* STIR-GUS-F2f7, (**b**) *Fno* Austria, (**c**) *Fno* PQ1104, (**d**) *Fno* Franc-COS1, and (**e**) *Fno* Ehime-1, showing dose-dependent reductions in larval survival. Heat-killed (HK) cells (equal dose to group injected with ca. 5 × 10^9^ CFU/mL; killed by 30 min at 90 °C) and sterile culture filtrates (CF) of each *Fno* isolate were also injected. Actual CFU/mL after plating bacterial suspensions of each *Fno* isolate on CHAH presented beside each line on the plots. Survival was 100% in the unmanipulated and PBS only control groups for each replicate (data not shown). *n* = 24

Having established that *Fno* STIR-GUS-F2f7 could cause mortality in *G. mellonella* larvae, the next experiment aimed to determine the virulence of five isolates of *Fno* obtained from separate disease outbreaks in fish. After injection into the larvae of different doses of each isolate (ca. 1 × 10^8^, 5 × 10^8^, 1 × 10^9^ or 5 × 10^9^ CFU/mL), in each case there was a dose-dependent reduction in larval survival, with injection of greater CFU/mL causing greater reductions in larval survival (Fig. 2). For each *Fno* isolate, the area under each curve was determined for each dose of CFU/mL and a cumulative value calculated. Accordingly, the most to least virulent *Fno* isolate in the larvae was of the order: Austria > PQ1104 > Franc-COS1 > STIR-GUS-F2f7 > Ehime-1. Heat-killed cells of each *Fno* isolate caused some mortality in the larval groups, but typically survival was reduced to an extent similar to injection with 10 to 50 times fewer live cells (Fig. 2), indicating live bacteria to be far more capable of exploiting the larval host, probably through the production of virulence factors, and larvae were not dying solely due to toxicity associated with injection with a large abundance of *Fno* cells. Larvae injected with heat-killed cells darkened almost immediately after injection, suggesting rapid immune recognition of pathogen-associated molecular patterns and possible masking and evasion of recognition by living *Fno* cells. Melanisation of larvae occurred more quickly and extensively with increasing doses of each *Fno* isolate, though injection of culture filtrate led to minimal changes in body colour. Interestingly, in the case of *Fno* Austria and *Fno* PQ1104 (the two most virulent isolates), survival of larvae injected with sterile culture filtrate led to reductions in survival similar to injection with ca. 1 × 10^8^ CFU/mL of live bacteria (Fig. 2), perhaps indicating the production of extracellular virulence factors by these isolates *in vitro*.

### Enumeration of *Fno* in *G. mellonella* after injection

**Fig. 3:**
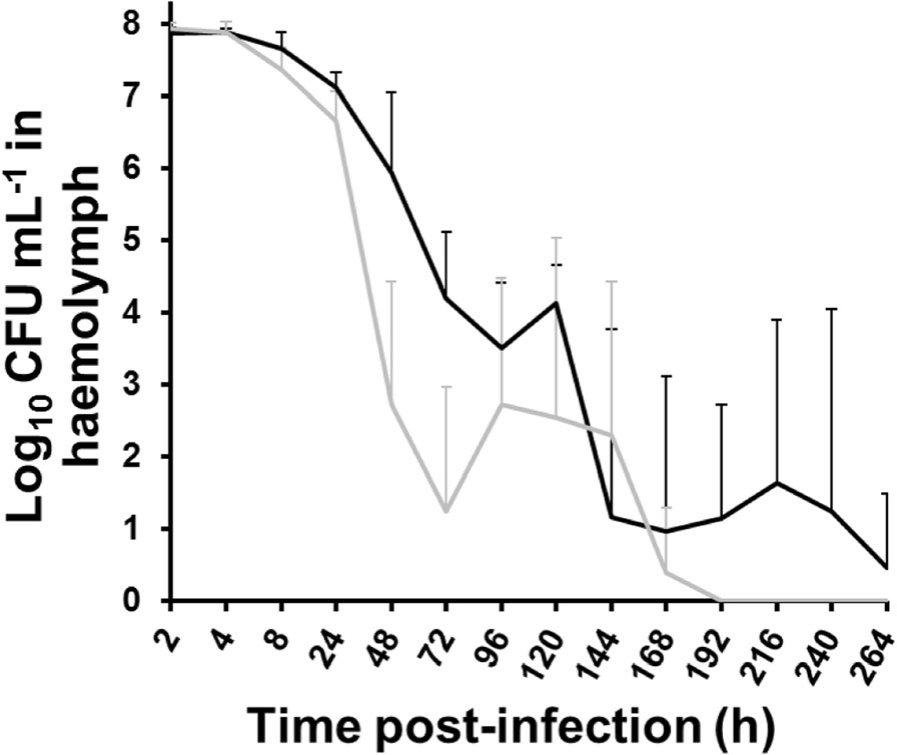
Enumeration of *Fno* in *Galleria mellonella* larva after injection. Abundance of *Fno* STIR-GUS-F2f7 (black line) and *Fno* Ehime-1 (grey line) in the haemolymph of *G. mellonella* larvae during 264 h at 28 °C after injection with ca. 1 × 10^9^ CFU/mL. Actual CFU/mL after plating bacterial suspensions of each *Fno* isolate on CHAH were: 5.7 × 10^9^ for *Fno* STIR-GUS-F2f7 and 1.6 × 10^9^ for *Fno* Ehime-1. Unmanipulated and PBS only larvae were sampled at the start, middle (144 h) and end of the experiment and no *Fno* colonies were recovered (data not shown). Bars are means of log_10_ transformations of (CFU/mL + 1) data + one standard deviation (*n* = 5)

Abundance of *Fno* in the haemolymph of *G. mellonella* larvae was assessed after injection with 1 × 10^9^ CFU/mL of *Fno* STIR-GUS-F2f7 or *Fno* Ehime-1 isolate by collecting the haemolymph and plating on cysteine heart agar (Melford Laboratories Ltd., Ipswich, UK) supplemented to 10% bovine haemoglobin solution (Becton Dickenson BBL, Sparks, MD, USA) to give CHAH medium and containing for this experiment 1 mg/L penicillin and 1 mg/L amphotericin B. While *Fno* CFU in the haemolymph reduced during the 264 h incubation for both isolates, it was the less virulent *Fno* Ehime-1 isolate that reduced in abundance more quickly than the *Fno* STIR-GUS-F2f7 isolate; indeed, *Fno* Ehime-1 was not detected at or after 192 h (Fig. 3). No *Fno* colonies were recovered from PBS only or unmanipulated groups of *G. mellonella* larvae.

### Rescue of *G. mellonella* from lethal dose of *Fno* by antibiotic therapy

**Fig. 4:**
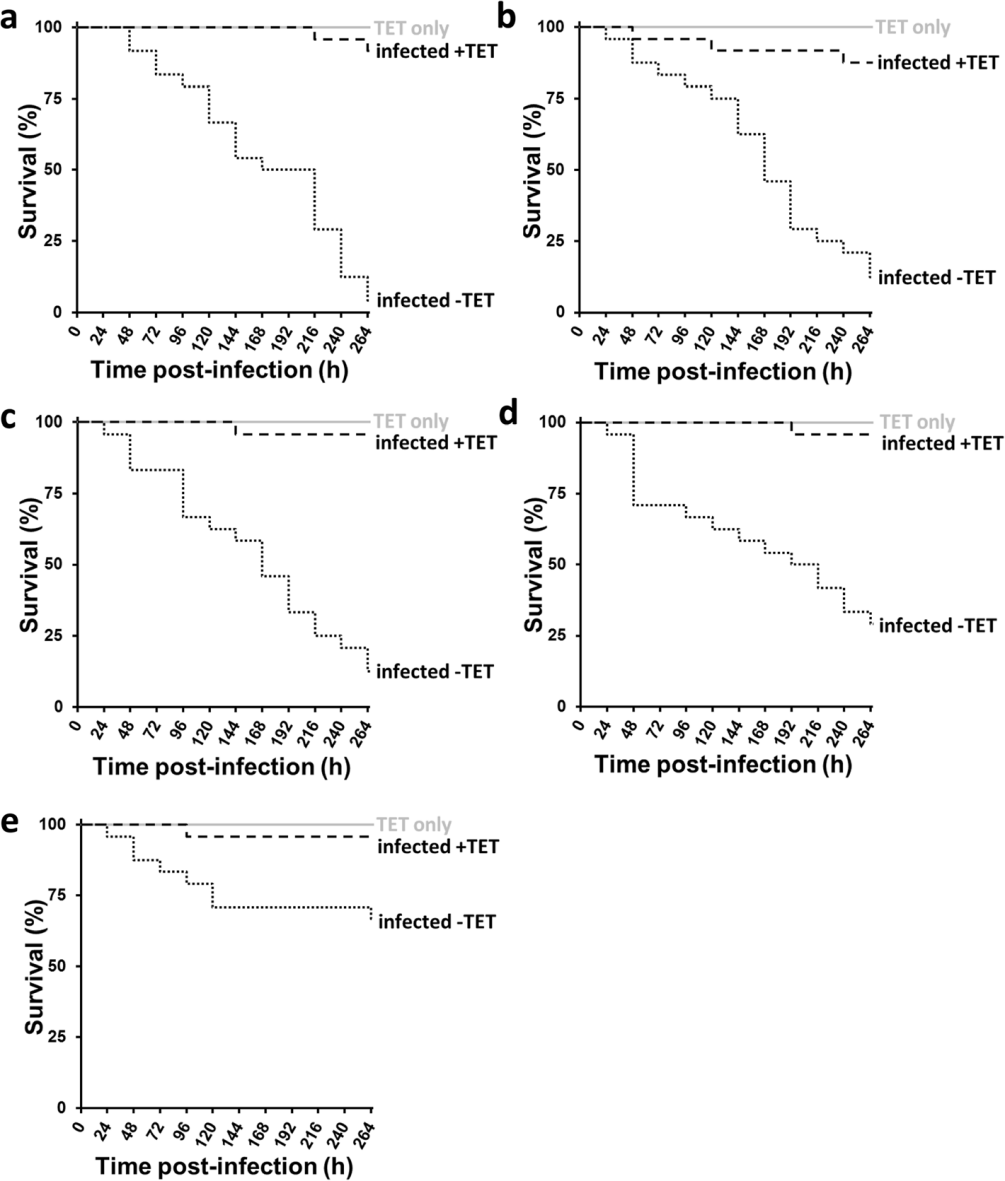
Effect of antibiotic therapy on *Galleria mellonella* larva survival after injection with *Fno*. Kaplan-Meier plots of *G. mellonella* larva survival during 264 h at 28 °C after injection of ca. 1 × 10^9^ CFU/mL of (**a**) *Fno* STIR-GUS-F2f7, (**b**) *Fno* Austria, (**c**) *Fno* PQ1104, (**d**) *Fno* Franc-COS1, and (**e**) *Fno* Ehime-1, and treatment with tetracycline at 10 mg/g body weight at 2, 24 and 48 h (infected +TET), showing that the antibiotic treatment increased larval survival compared to groups treated with PBS (infected -TET). Actual CFU/mL after plating bacterial suspensions of each *Fno* isolate on CHAH were: 0.81–1.33 × 10^9^ for *Fno* STIR-GUS-F2f7, 0.81–1.25 × 10^9^ for *Fno* Austria, 0.90–1.10 × 10^9^ for *Fno* PQ1104, 1.32–1.35 × 10^9^ for *Fno* Franc-COS1, and 0.87–0.90 × 10^9^ for *Fno* Ehime-1. One control group of larvae was injected with PBS instead of bacteria and treated with the tetracycline (TET only) to assess the toxicity of the antibiotic alone, while survival was 100% in the unmanipulated and PBS only control groups for each replicate (data not shown). *n* = 24

Larvae injected with lethal doses of each of the five *Fno* isolates (ca. 1 × 10^9^ CFU/mL) were treated with three doses of tetracycline (10 mg/g body weight at 2, 24 and 48 h post-infection) and in each case antibiotic treatment led to significant (*p* < 0.05) increases in larval survival, indicating that infections leading to mortalities could be prevented through antimicrobial therapy (Fig. 4).

### Histology of *Fno* infection of *G. mellonella*

**Fig. 5:**
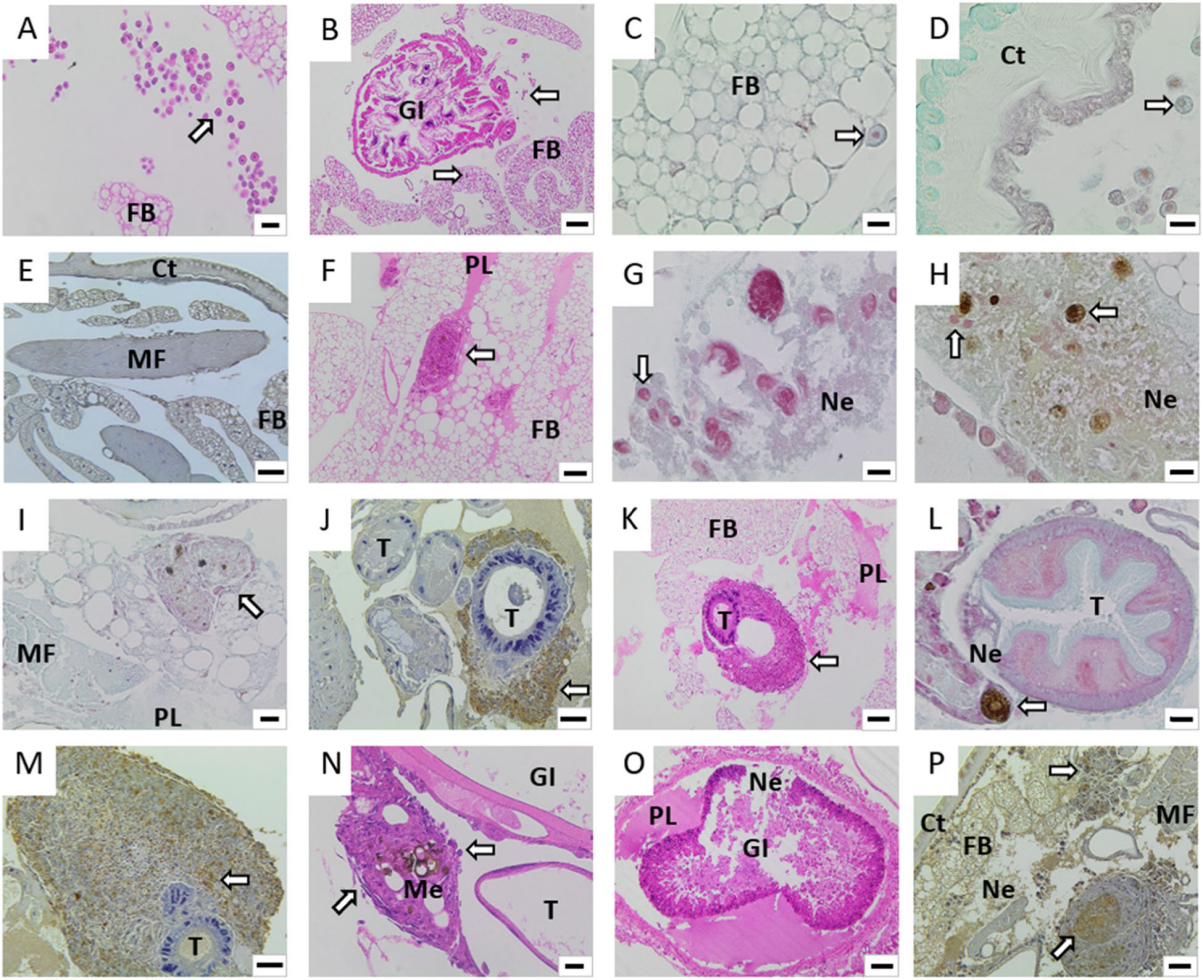
Progression of infection in *Galleria mellonella* larva tissues after injection with *Fno*. Visualisation of *G. mellonella* larva tissues during 96 h after injection of *Fno* STIR-GUS-F2f7 in 10 μL phosphate-buffered saline at 1 × 10^9^ CFU/mL and incubation at 28 °C. Tissues were stained by haematoxylin and eosin (**a**, **b**, **f**, **k**, **n**, **o**), Gram Twort (**c**, **d**, **g**, **h**, **i**, **l**) or immunohistochemistry (IHC) with anti-*Fnn* primary antibodies that cross-react with *Fno* (**e**, **j**, **m**, **p**) in unmanipulated control larvae at 0 h (**a-e**) or larvae injected with *Fno* and sampled at 48 h (**f-j**), 72 h (**k-h**) and 96 h (**n-p**). Control larvae at 0 h showed scattered haemocytes in and around the fat body (**a-c**), subcuticular area (**d**) and in clusters surrounding the gastrointestinal tract (**b**); *Fno* was not detected by IHC (**e**). At 48 h, larvae injected with *Fno* showed infiltration of haemocytes into the fat body, eosinophilic fluid in the coelomic cavity (**f**) and enlarged haemocytes containing bacteria (**g**, **h**); melanised haemocytes were also observed (**h**). Clusters of haemocytes formed nodules, often surrounded by flattened cells (**i**), and *Fno* was detectable by IHC (**j**). At 72 h, large nodules had formed (**k**), and enlarged and melanised haemocytes were observed (**l**); great abundances of *Fno* cells were detected by IHC (**m**, **p**). At 96 h, large and increasingly melanised nodules were observed, while haemocytes at the periphery were flat in appearance (**n**); there was evidence for the recruitment of new, round haemocytes (**n**). Large protein lakes and severe tissue necrosis were observed (**o**) and great abundances of *Fno* cells were detected by IHC (**p**). Ct, cuticle; FB, fat body; GI, gastrointestinal tract; MF, muscle fibres; Me, melanin; Ne, necrosis; PL, protein lake; T, trachea. Scale bars: a, i = 20 μm; b, k, o = 100 μm; c, d, g, h, l = 10 μm; e, f, j, n, p = 50 μm

Histological analyses revealed the progression of infection in the larval tissues and the larva immune response (Fig. 5). In control larvae, a few scattered haemocytes were located in and around the normal fat body (Fig. 5a-c), around the muscle fibres and tracheal walls, circulating in the haemolymph, in the subcuticular area (Fig. 5d) and in small clusters surrounding the gastrointestinal tract (Fig. 5b). There was no evidence for *Fno* in any control tissues by immunohistochemistry (IHC) when performed with polyclonal anti-*Fnn* NCIMB 14265 antibodies that cross-react with *Fno* (Fig. 5e). In larvae injected with 1 × 10^9^ CFU/mL of *Fno*, at 48 h haemocytes had infiltrated the fat body (Fig. 5f), while the presence of eosinophilic fluid in the coelomic cavity suggested vascular leakage and the mounting of an inflammatory response (Fig. 5f). Enlarged haemocytes containing Gram-negative bacteria (Fig. 5g) and melanised haemocytes were observed in tissues, particularly within the fat body where necrosis was also evident (Fig. 5h), and this was consistent with the timing of the darkening of the larval body observed in earlier experiments. Larger clusters of haemocytes formed distinct nodules, often surrounded by flattened cells exhibiting the spindle morphology (Fig. 5i). *Fno* was detected by IHC in the subcuticular area, the gastrointestinal tract and at the tracheal walls (Fig. 5j).

At 72 h, greater abundances of haemocytes and the formation of large nodules were observed in the subcuticular area, muscle fibres, fat body and tracheal walls (Fig. 5k). Enlarged and melanised haemocytes were observed in various tissues, including around the trachea (Fig. 5l). Great abundances of *Fno* cells were detected by IHC in the fat body, muscle fibres, subcuticular areas and trachea (Fig. 5m). At 96 h, multiple large and increasingly melanised nodules were observed (Fig. 5n), which was consistent with progressive darkening of larval body observed macroscopically. Haemocytes at the periphery of nodules were flat in appearance and there was evidence for recruitment of new, round haemocytes to the nodules (Fig. 5n). Large protein lakes and severe tissue necrosis were evident, especially around the tracheal walls and gastrointestinal tract, where necrosis was extensive (Fig. 5o). *Fno* was detected by IHC in great abundance inside and around the gastrointestinal tract, tracheal walls and fat body (Fig. 5p).

### Virulence of *Fno* isolates in *O. niloticus*

**Fig. 6:**
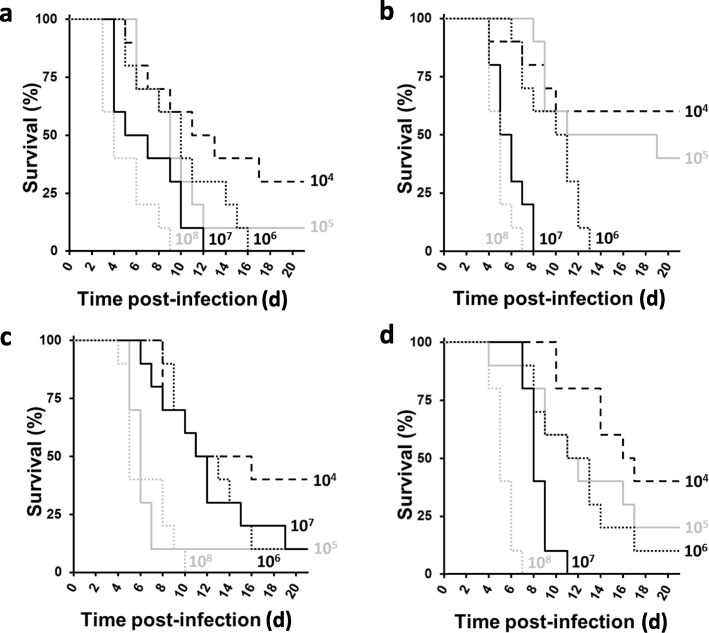
Effect of different doses of *Fno* on *Oreochromis niloticus* survival. Kaplan-Meier plots of *O. niloticus* survival during 20 d at 23 ± 2 °C after intraperitoneal injection of 100 μL of *Fno* suspensions at ca. 1 × 10^4^, 1 × 10^5^, 1 × 10^6^, 1 × 10^7^ and 1 × 10^8^ CFU/mL: (**a**) *Fno* STIR-GUS-F2f7, (**b**) *Fno* Austria, (**c**) *Fno* PQ1104, and (**d**) *Fno* Franc-COS1, showing dose-dependent reductions in fish survival. No mortalities were observed in the PBS only control group. *n* = 20

In a final experiment, the virulence of each of four *Fno* isolates in *O. niloticus* was assessed by intraperitoneal injection of 100 μL of ca. 1 × 10^4^, 1 × 10^5^, 1 × 10^6^, 1 × 10^7^ or 1 × 10^8^ CFU/mL and monitoring the fish for 20 d at 23 ± 2 °C. *Fno* was detected in each dead/moribund fish. Consistent with the *G. mellonella* larva findings, for each *Fno* isolate there was dose-dependent reductions in fish survival, with injection of greater CFU/mL causing greater group mortalities (Fig. 6). For each *Fno* isolate, the area under each curve was determined for each dose of CFU and a cumulative value calculated. Accordingly, the most to least virulent *Fno* isolate in the tilapia was of the order: STIR-GUS-F2f7 > Austria > PQ1104 > Franc-COS1. *Fno* Ehime-1 was not tested in the fish as this isolate was non-virulent in the pre-challenge test (data not shown).

## Discussion

Francisellosis is an emerging bacterial disease in tilapia farming caused by *Fno* and relatively little is known of the infection biology of this bacterium, with efforts towards improved understanding hampered by difficulties associated with performing fish trials, including cost, legislative burden and ethical acceptability. Non-vertebrate alternative hosts offer solutions to many of these problems and can deliver valuable insights in host-pathogen interactions given the similarities in host innate immunity due to the universal ancestry of all organisms. *G. mellonella* is an alternative host used widely for understanding virulence and pathogenicity of bacterial pathogens, including those causing disease in fish [23], due to a range of benefits around ease of use, ability to examine pathology and availability of the genome sequence [36, 44]. Hence, this present study aimed to assess whether *G. mellonella* would be suitable for studying *Fno* infections.

Direct injection of *Fno* into the *G. mellonella* larva appeared to cause infection, as evidenced by the far greater mortalities caused by living bacteria compared to heat-killed counterparts; the dose-dependent increase in mortalities caused by greater doses of *Fno*; and significant improvement in larval survival after treatment with an antibiotic to which *Fno* was susceptible. Antibiotic therapy kills or inhibits the replication of the bacterium and allows the host immune system to counter successfully this microbial invasion. Importantly, there was good correlation in the relative virulence of four *Fno* isolates between *G. mellonella* and *O. niloticus* hosts, although *Fno* STIR-GUS-F2f7 did differ in virulence between the two hosts. This finding suggests similar virulence factors are involved in insect and fish infections, though this needs to be confirmed by further approaches such as testing of knockout strains, and these investigations might uncover the reason for the discrepancy between hosts in the virulence of *Fno* STIR-GUS-F2f7. Still, *Fno* STIR-GUS-F2f7 was detected intracellularly in *G. mellonella*, as observed with other *Francisella* spp. in this insect model [43], and this pathogen is known to survive intracellularly in fish host cells [7–9, 11, 45], which further supports *G. mellonella* to be a suitable alternative host for studying *Fno* infections. The progression of *Fno* infection in *G. mellonella* is similar in nature to previous findings where these larvae have been inoculated with other pathogens, with evidence of non-host recognition, an inflammatory response, the formation of melanised nodules and tissue necrosis [46–49]. Heat-killed *Fno* were recognised by the insect and mounted an immune response, as evidenced by the darkening of the larval colour shortly after injection due to activation of prophenoloxidase pathway leading to melanisation [28]. Mortalities did occur in the heat-killed bacteria groups, which is probably due to the presence of a large abundance of elicitors being recognised as foreign by the host and the stress associated with the host mounting a massive immune response.

*Fno* appeared to cause greatest mortality in *G. mellonella* at 28 °C, which is close to the optimum for replication of this bacterium *in vitro* [8, 50, 51]. In farm conditions, *Fno* typically causes infections in tilapia when the water temperature reduces and this becomes more prevalent below 25 °C, probably due to increased host stress [1, 52], which may explain the disparity between the models because *G. mellonella* typically lives around 28 °C in its natural environment and thus is probably in better physiological condition [53]. Nevertheless, the *G. mellonella* model provides an opportunity to investigate the temperature-regulation of virulence factor expression in *Fno* more ethically, as it can be incubated at a range of temperatures [24, 25] and quantitative polymerase chain reaction (PCR) can be performed to quantify bacterial gene expression in vivo [54].

Relatively little is known of the key virulence factors involved in *Fno* infection, though oxidative stress response proteins (e.g., Hsp60, Hsp90), type-4 pili, iron sequestration mechanisms and a type-VI secretion system have been detected in the *Fno* genome, all of which are key virulence factors for other pathogenic *Francisella* spp. [7, 55–57]. Even so, there is much to be done to uncover the suite of virulence factors important for *Fno* infection and certainly *G. mellonella* lends itself to high-throughput screens, which are often necessary for these types of studies. Moreover, the detection of lethal activity in sterile culture filtrates in this present study suggests the presence of extracellular virulence factors, such as toxins or degradative enzymes, and fish-pathogenic *Francisella* spp. do produce outer-membrane vesicles embedded with virulence factors [45, 58]. Beyond virulence factor discovery and probing of host-pathogen interactions, the *G. mellonella* system would be useful for determining the relative virulence of isolates, as such information is useful for identifying particularly problematic (i.e., virulent) strains.

Interestingly, when haemolymph was collected from inoculated larvae and plated on to agar, *Fno* did not appear to replicate, which is in contrast to a previous study on *V. anguillarum* where more virulent isolates replicated inside the haemolymph to a far greater extent than less virulent isolates [23]. However, unlike McMillan et al. [23], where *V. anguillarum* was confirmed to be mostly in the haemolymph compared to the rest of the body, it was not possible to obtain *Fno* CFU counts for whole-larva homogenate, due to the difficulty of selecting for this fastidious bacterium against the abundant bacteria found on the larva surface and in the gastrointestinal tract (data not shown). Nevertheless, the histopathological analyses appeared to support replication of *Fno* inside the *G. mellonella* as the bacteria appeared to become more abundant during the progression of the infection, though the *Fno* were increasingly detected inside haemocytes, in tissues including the fat body, or in aggregates in the haemolymph, all of which might explain the lower than expected *Fno* CFU counts in the haemolymph when plated on agar. Indeed, the protocol in this present study was refined to lyse the host cells in the haemolymph because this increased *Fno* CFU abundance (data not shown), likely by releasing the bacterium from the confines of host cells. Hence, agar counts are probably not a true representation of *Fno* replication in the larvae and quantification of *Fno* abundance during infection from pathology preparations, or by molecular methods such as quantitative PCR, likely offer more accurate estimates.

## Conclusion

In conclusion, this present study demonstrates *G. mellonella* to be a useful model for studying infections caused by *Fno* and thus can be applied to increase our understanding of the virulence and pathogenicity of this pathogen. Such an approach will support efforts towards solutions that prevent and reduce outbreaks of francisellosis in tilapia and improve production in this important industry.

## Methods

### Reagents

Unless stated, all reagents were sourced from Sigma Aldrich Ltd. (Poole, United Kingdom [UK]), while solvents were purchased from Thermo Fisher Scientific (Loughborough, UK). One-litre of PBS (0.02 M, pH 7.2) consisted 0.876 g NaH_2_PO_4_.2H_2_O (VWR International Ltd., Lutterworth, UK), 2.56 g Na_2_HPO_4_.2H_2_O (VWR International Ltd) and 8.77 g NaCl. Culture media, PBS and water were sterilised by autoclaving at 121 °C for at least 15 min. Antibiotic solutions were sterilised by passing through a sterile polyethersulfone 0.22-μm filter (Millipore, Watford, Herts, UK).

### Bacteria and culture conditions

Five isolates of *Fno* were collected from separate outbreaks of francisellosis: Austria (isolated from ornamental Malawi cichlids [59]), Ehime-1 (DSM 21254, type strain; isolated from three-lined grunt, *Parapristipoma trilineatum*, in Japan in 2001 [51]), Franc-COS1 (isolated from *Oreochromis* sp. in Mexico in 2012 [60]), PQ1104 (isolated from *Oreochromis* sp. in Costa Rica in 2007), and STIR-GUS-F2f7 (isolated from *O. niloticus* in UK in 2012 [10]). Routinely, *Fno* was cultured at 28 °C on CHAH medium or in cation-adjusted Mueller-Hinton II broth (MHB; Becton Dickinson BBL, Sparks, MD, USA) supplemented with 0.1% glucose and 2% IsoVitaleX (Becton Dickinson BBL). Glycerol stocks (20%) were prepared for long-term storage at − 70 °C. The bacterial isolates were confirmed as *Fno* according to the methods described by Frerichs and Millar [61], including primary identification tests (Gram-staining, catalase, oxidase, oxidation/fermentation of glucose, and motility) and biochemical profiles determined with API20E and ZYM kits (BioMerieux; Marcy L’étoile, France) according to the manufacturer guidelines except the inoculated strips were incubated at 28 °C and read at 72 h and 24 h, respectively.

### *G. mellonella*

Final instar stage *G. mellonella* larvae were purchased from UK Waxworms Ltd. (Sheffield, UK). Moribund, discoloured and dead larvae were removed and only those with uniform cream colouration and of 250–350 mg were used for experiments. Routinely, larvae were kept in the dark in Petri dishes at 4 °C and used within one week of receipt.

### Inoculum preparation

A few colonies of *Fno* were inoculated into 15 mL supplemented MHB and cultured for 20 h at 150 rpm to mid-logarithmic phase of growth. Bacterial cells were harvested by centrifugation (3000×g, 15 min, 4 °C) and then washed twice by resuspension in 10 mL PBS, before finally re-suspending in 15 mL PBS. Cell density was determined by measuring absorbance at 600 nm (A_600_) using a spectrophotometer (Cecil CE-2014; Buck Scientific, Inc., East Norwalk, CT, USA) and then adjusted by dilution with PBS to the desired CFU/mL according to a standard curve (data not shown). Typically, serial 10-fold dilutions of bacterial suspensions in PBS were plated on CHAH (6× 20 μL of each dilution) to determine accurate CFU/mL after incubation (48 h, 28 °C), or for fish trials by the drop plate method as described by Chen et al. [62].

### Injection of *G. mellonella* larvae

*G. mellonella* larvae experiments were performed in a bacteriological laboratory according to the methods described by McMillan et al. [23]. Briefly, larvae were injected with 10 μL of solution (bacterial suspension, antibiotic or PBS) using a 50-μL Hamilton syringe (Sigma Aldrich Ltd) into the haemocoel via the last left pro-leg, after the larvae had been cooled on ice for 5 min. Consecutive washes of 1% (w/v) sodium hypochlorite solution, 70% ethanol and sterile water were used to clean the syringe in between experimental groups. After injection, each group of larvae was kept in a disposable 90-mm diameter plastic Petri dish and incubated in the dark for 264 h at 28 °C, unless stated. The larvae were assessed each 24 h for survival and were considered dead (and removed from the Petri dish) if displaying no response to the tactile stimulus given by brushing with a sterile inoculation loop. Each experimental group consisted 12 larvae selected at random and each experiment was repeated using larvae from a different batch to give *n* = 24, with mean percentage group survival calculated prior to preparing Kaplan-Meier plots. Two control groups were included in each experiment: one group of *G. mellonella* larvae received injections of ‘PBS only’ to assess the impact of physical trauma, while a second ‘unmanipulated’ group received no injections and was used to assess background larval mortality.

### Effect of temperature on *G. mellonella* larvae survival after injection with *Fno*

Groups of *G. mellonella* larvae were injected with a ca. 1 × 10^9^ CFU/mL suspension of *Fno* STIR-GUS-F2f7 and incubated at 15, 22, 25, 28 or 37 °C for 264 h to determine the effect of temperature on larval survival after injection with live *Fno*. This experiment was performed only once.

### Virulence of different *Fno* isolates in *G. mellonella*

Groups of *G. mellonella* larvae were injected separately with ca. 1 × 10^8^, 5 × 10^8^, 1 × 10^9^ or 5 × 10^9^ CFU/mL suspensions of each of the *Fno* isolates. In addition, the supernatant from the first centrifugation step to harvest the *Fno* cells (see ‘Inoculum preparation’) was passed through a sterile polyethersulfone 0.22-μm filter to give sterile culture filtrates. Sterile culture filtrates were also injected into groups of *G. mellonella* larvae, as this can indicate the presence of extracellular virulence factors such as toxins and enzymes. Finally, PBS-washed suspensions of each *Fno* isolate at ca. 5 × 10^9^ CFU/mL were heat-killed for 30 min at 90 °C and administered to further groups of *G. mellonella* larvae. Heat-killing was confirmed by the absence of colonies on CHAH inoculated with 100 μL of bacterial suspension and incubated for 48 h at 28 °C.

### Enumeration of *Fno* in *G. mellonella* larvae haemolymph

To assess the abundance of *Fno* in the haemolymph of *G. mellonella* larvae after injection, groups of 175 *G. mellonella* larvae were injected with ca. 5 × 10^8^ CFU/mL of *Fno* STIR-GUS-F2f7 or *Fno* Ehime-1 and incubated as above; more larvae were injected than would be required to ensure there would be sufficient surviving larvae to sample at each intended time point. Five surviving larvae in each group were selected at random for determination of bacterial load at 2, 4, 8 and 24 h, and then every 24 h up to 264 h. Prior to sampling, larvae were cooled on ice for 30 min and then the body surface was sterilized by spraying with 70% ethanol and wiping with sterile tissue paper. The last abdominal segment (final 2 mm of the body) was removed aseptically with sterile scissors and harvesting the haemolymph according to McMillan et al. [23]. The haemolymph (ca. 5–10 μL) was drained from each larva into a sterile 0.5-mL micro-centrifuge tube, and then pipetted up and down 30 times before briefly agitated on a vortex to lyse the cells (modified from Senior et al. [63]). Ten-fold serial dilutions were performed in PBS in sterile 96-well microtitre plates, before 10 μL of each dilution was plated on CHAH supplemented with 1 mg/L penicillin and 1 mg/L amphotericin B to select for *Fno* and against other bacteria. Still, primary identification tests (see ‘Bacteria and culture conditions’) were performed on a subset of colonies to confirm that those recovered from infected larvae were indeed *Fno*. Haemolymph was also collected from PBS only and unmanipulated control groups at the start, middle (144 h) and end of the experiment.

### Antibiotic treatment of *G. mellonella* larvae injected with *Fno*

To assess whether antibiotic therapy would rescue *G. mellonella* larvae from lethal doses of each of the *Fno* isolates (ca. 1 × 10^9^ CFU/mL), sterile-filtered tetracycline in PBS (ca. 10 mg/g body weight) was administered by injection at 2, 24, and 48 h post-infection. Each *Fno* isolate had been shown to be highly susceptible to the action of tetracycline by disk diffusion (data not shown). In addition to the PBS injected and unmanipulated controls, two extra control groups were prepared: one group of *G. mellonella* larvae was injected with PBS instead of bacteria and then with tetracycline (to assess the toxicity of the antibiotic), and another group was injected with bacteria and then with PBS instead of tetracycline (to confirm the virulence of the *Fno*). The multiple injections were administered to different prolegs, as described in Desbois and Coote [64].

### Histopathology and localisation of *Fno* in *G. mellonella* larvae cells

To observe the progression of infection and localise *Fno* STIR-GUS-F2f7 in the *G. mellonella* larvae tissues, 20 *G. mellonella* larvae were injected with 1 × 10^9^ CFU/mL and incubated as above. Three larva were sampled at 48, 72 and 96 h for histopathological analyses by haematoxylin and eosin (H & E) staining, Gram Twort staining and IHC. Each larva was anesthetized on ice for at least 30 min, injected with ca. 100 μL of 10% (v/v) neutral-buffered formalin and then kept in this solution for 24 h at 4 °C to fix the internal organs and block melanisation [65]; unmanipulated larvae at 0 h were sampled as controls.

#### Tissue sectioning

Tissue sections were prepared from whole larvae that were dissected transverse to the median plane of the body into six equal sections using a scalpel (i.e., one distal, four middle and one proximal), and then each section was wrapped in biopsy tissue paper prior to placing into standard tissue cassettes for processing overnight on a processor (Shandon Citadel 2000; Thermo Fisher Scientific) and subsequent embedding in paraffin wax (EG1160 Histoembedder; Leica Biosystems, Nussloch, Germany). The procedure was performed carefully to avoid squeezing the larval tissues. Each wax block was trimmed with a microtome (RM 2255; Leica Biosystems) to expose the tissue and soaked in water for 30 min prior to cutting. Four micrometer-thick sections were mounted onto glass slides (Solmedia Supplying Science, Shrewsbury, UK) and dried in an oven overnight at 60 °C. Then the sections were deparaffinised in xylene for 3 min then 2 min (twice), rehydrated in absolute ethanol (2 min) and methylated spirit (1 min), before rinsing in tap water (1 min).

#### H & E staining

Sections were stained with Mayer’s haematoxylin ‘Z’ stain (CellPath Ltd., Newtown, UK) for 5 min and then rinsed in tap water. Next, the sections were dipped three times in 1% acid alcohol (methylated spirit:hydrochloric acid; 100:1), rinsed in tap water, counterstained with eosin solution (1% [w/w] eosin Y:Putt’s eosin [Cellpath, Newton, UK]; 8:1) before rinsing again in tap water. The slides were dehydrated in absolute ethanol for 2 min then 1 min (twice), before being cleared with xylene (5 min) and mounted with Pertex medium (HistoLab Products Ab, Gothenburg, Sweden). Once dry, the slides were examined using an upright light microscope (BX53M; Olympus, Southend-on-Sea, UK) and images were collected with a digital camera (SC100; Media Cybernetics, Abingdon, UK) and cellSens 1.17 software (Olympus).

#### Gram Twort staining

Sections were stained with 2% Lillie’s crystal violet solution (500 mL consists 10 g crystal violet [Merck Chemical, Darmstadt, Germany] and 4 g ammonium oxalate in 20% ethanol) for 1 min and then rinsed in running tap water. The slides were treated with 0.4% Lugol’s iodine solution (100 mL consists 1 g iodine [Thermo Fisher Scientific] and 2 g potassium iodide [VWR International Ltd] in water) for 1 min, before being rinsed in tap water, and flooded with acetone (Thermo Fisher Scientific) for 2–5 s. The slides were rinsed again in running water and counterstained with Twort’s stain in a closed Wheaton Coplin staining jar (S5516-6EA; Sigma Aldrich Ltd) for 5 min. Five hundred millilitres of Twort’s stain consisted 100 mg of neutral red and 900 mg of 0.2% fast green (Thermo Fisher Scientific) in 95% ethanol, with a working solution prepared by diluting this stock solution in distilled water (1:3). After staining, the slide was rinsed in tap water. Finally, each section was rapidly dehydrated by dipping twice in absolute ethanol for 5 s each time, and then cleared, mounted and examined as described in Section 5.10.2.

#### IHC

IHC was performed to localise *Fno* in larvae tissues using polyclonal anti-*Fnn* NCIMB 14265 antibodies that cross-react with *Fno*. First, sections were pre-treated with 3% (v/v) hydrogen peroxide in methanol for 10 min to block endogenous peroxidase activity and then washed thrice in PBS. All incubations were performed in a humidified chamber at room temperature (ca. 22 °C). Non-specific binding of the secondary antibody was blocked by incubation with normal goat serum (Sigma Aldrich Ltd., UK) diluted 1:10 in PBS for 15 min. The serum was discarded, slides tapped dry and then rabbit antisera containing the primary antibodies (diluted 1:300 in PBS) was added to the slides and incubated for 1 h (PBS was added in place of antisera in a negative control). The slides were washed with PBS and then goat anti-rabbit immunoglobulin G conjugated to horseradish peroxidase conjugate (1:200; Sigma Aldrich Ltd) was added for 30 min. Slides were washed in PBS and incubated with the Immpact DAB peroxidase substrate (Vector Laboratories, Peterborough, UK) for 10 min, before the reaction was stopped by immersion in tap water. The slides were counterstained with Mayer’s haematoxylin for 4 min, rinsed in tap water, dehydrated in a graded series of ethanol (70% followed by 100% for 5 min each) and cleared in xylene (5 mins twice) prior to adding a coverslip with Pertex mounting media. Slides were examined by light microscopy.

### Virulence of different *Fno* isolates in tilapia

#### Fish and rearing conditions

Red Nile tilapia (*O. niloticus*) of 10 ± 0.5 g and 7.0 ± 0.19 cm were purchased from a private farm in Prachinburi, Thailand and transported to the research aquarium of Fish Vet Group Asia Ltd. (FVGAL), Chonburi, Thailand. Upon arrival, the fish were transferred to 100-L circular tanks in a recirculation system for acclimation. Water conditions were maintained as follows: 28 ± 1 °C; 6.5–7 mg/L dissolved oxygen; pH 7–7.5; ≤0.1 mg/L free ammonia; ≤0.25 mg/L nitrite; and ≤ 0.2 mg/L nitrate. Fish were acclimated for 2 weeks and fed at 3% body weight per day with a commercial tilapia feed (Charoen Pokphand Foods Public Company Ltd., Bangkok, Thailand). The *Fno*-free status of the fish was determined prior to challenge using samples of spleen and head kidney from four fish by bacteriological analyses and a *Francisella* genus-specific PCR performed as described previously [8, 66].

#### Fish challenge

First, each *Fno* isolate was passaged two times in three fish (each fish was ca. 20 g) by intraperitoneal (i.p.) injection of 10^10^ CFU/fish in PBS after being anaesthetised (stock of 10% benzocaine [w/w] prepared in 70% ethanol and used at 50 mL/L; Thermo Fisher Scientific), and then incubated for 4 d at 23 ± 2 °C. As *Fno* Ehime-1 did not cause any mortalities in either passage it was not included in the subsequent challenge trial (data not shown). Ten fish were allocated randomly into each of 40 3-L tanks containing 2.5 L of dechlorinated water and fish were not fed for 48 h prior to the *Fno* challenge. Tanks were divided into four main groups (one for each *Fno* isolate) and five sub-groups of duplicate tanks. Then the fish in each sub-group (*n* = 20) were i.p. injected separately with 100 μL of ca. 1 × 10^4^, 1 × 10^5^, 1 × 10^6^, 1 × 10^7^ or 1 × 10^8^ CFU/mL in PBS of each of the Fno isolates, with the bacterial inoculums prepared according to Section 5.4. A further two tanks with 10 fish in each contained the controls that received an injection of PBS only. Fish were maintained for 20 d at 23 ± 2 °C, fed *ad libitum* and examined four times per day for mortalities. To confirm recovery of *Fno* from dead and moribund fish, these animals were removed and tissues collected (including the head kidney and spleen) for: i) direct PCR with *Francisella* genus-specific primers (see ‘Fish and rearing conditions’); ii) isolation of bacteria on CHAH agar, followed phenotypic testing and PCR of colonies. Fish surviving to 20 d post-challenge were euthanised by overdose of anaesthetic (prepared and used as above) followed by a lethal blow to the head according to Schedule 1 technique of the UK Animals (Scientific Procedures) Act 1986.

### Data analyses

Where required, survival differences between groups were compared with the logrank test in Prism (GraphPad Software, San Diego, CA, USA) and a *p*-value of < 0.05 was considered to indicate a significant difference. Relative virulence of the *Fno* isolates in *O. niloticus* and *G. mellonella* models of infection were calculated using area under the curve of cumulative survival of each group of injected larvae and fish (as described in McMillan et al. [23]). For CFU over time data, means of log_10_ transformations of (CFU/mL + 1) were calculated and standard deviations determined.

## Data Availability

The datasets used and/or analysed during the current study are available from the corresponding author on reasonable request.

## Abbreviations

CFU: Colony-forming units
CHAH: Cysteine heart agar supplemented with 10% bovine haemoglobin solution
*Fnn*: *Francisella noatunensis* subsp. *noatunensis*
*Fno*: *Francisella noatunensis* subsp. *orientalis*
H & E: Haematoxylin and eosin
MHB: Mueller-Hinton II broth
PBS: Phosphate-buffered saline
UK: United Kingdom
